# Both-Column Acetabular Fractures: Does Surgical Approach Vary Based on Using Virtual 3D Reconstructions?

**DOI:** 10.3390/diagnostics13091629

**Published:** 2023-05-05

**Authors:** Judith F. Leemhuis, Nick Assink, Inge H. F. Reininga, Jean-Paul P. M. de Vries, Kaj ten Duis, Anne M. L. Meesters, Frank F. A. IJpma, on behalf of the Pelvic Fracture Consortium

**Affiliations:** 1 Department of Surgery, University Medical Center Groningen, University of Groningen, 9713 GZ Groningen, The Netherlands; 2 3D Lab, University Medical Center Groningen, University of Groningen, 9713 GZ Groningen, The Netherlands

**Keywords:** acetabular fracture, acetabulum, both column, 3D, three-dimensional, survey, surgical approach

## Abstract

Displacement of the anterior and posterior column complicates decision making for both-column acetabular fractures. We questioned whether pelvic surgeons agree on treatment strategy, and whether the use of virtual 3D reconstructions changes the treatment strategy of choice. A nationwide cross-sectional survey was performed in all pelvic trauma centers in the Netherlands. Twenty surgeons assessed 15 both-column fractures in 2D as well as 3D. Based on conventional imaging, surgical treatment was recommended in 89% of cases, and by adding 3D reconstructions this was 93% (*p* = 0.09). Surgical approach was recommended as anterior (65%), posterior (8%) or combined (27%) (poor level of agreement, κ = 0.05) based on conventional imaging. The approach changed in 37% (*p* = 0.006), with most changes between a combined and anterior approach (still poor level of agreement, κ = 0.13) by adding 3D reconstructions. Additionally, surgeons’ level of confidence increased from good in 38% to good in 50% of cases. In conclusion, surgeons do not agree on the treatment strategy for both-column acetabular fractures. Additional information given by 3D reconstructions may change the chosen surgical approach and increase surgeons’ confidence about their treatment decision. Therefore, virtual 3D reconstructions are helpful for assessing both-column fracture patterns and aid in the choice of treatment strategy.

## 1. Introduction

Both-column acetabular fractures are defined as complete articular fractures involving the anterior as well as the posterior column of the pelvis [1]. These fractures are considered extensive, multi-fragmentary, and challenging to treat [2]. The quality of fracture reduction is associated with clinical outcome in acetabular fracture surgery [3]. Acetabular fractures can lead to severe post-traumatic arthritis, with the subsequent need for conversion to total hip arthroplasty, when not treated properly [2]. Mears et al. [3] found that both-column fractures have a significantly lower rate of peri-operative anatomical reduction than other types of acetabular fractures. The quality of reduction relies on both predetermined factors, such as age, fracture type, and comorbidity, and on controllable factors, such as surgical approach [4].

No single surgical approach can expose both columns of the acetabulum in the case of both-column acetabular fractures. Only a few guidelines [5] have been proposed on which surgical approach to use for different fracture patterns. Moreover, these guidelines are based on experts’ opinions and thus a low level of evidence. No scientific evidence exists on how to decide on the best surgical exposure for these fractures. Thus, decisions regarding surgical approach for treating both-column fractures are mainly based on the clinical experience of the surgeon and surgical approach may vary substantially between surgeons. Suggestions are that a merely anterior displaced fracture could sufficiently be reduced through an anterior approach: i.e., a Modified Stoppa, Pararectus, or Ilioinguinal approach [1,5,6]. For mainly posterior displaced fractures, a posterior approach, such as the Kocher–Langenbeck approach, would be sufficient. A sequential approach might be used, i.e., a posterior approach combined with an anterior approach, when a single approach cannot sufficiently reduce the fracture. The decision of which surgical approach to use is an important part of preoperative planning because each approach requires its own surgical preparation (e.g., patient position on the table, surgical instruments, position of the preferred intraoperative imaging modality).

Currently, preoperative planning is mainly based on pelvic radiographs and computed tomography (CT) scans. However, the complex three-dimensional (3D) shape of the pelvis and acetabulum can make it difficult to obtain a full understanding of the fracture lines based on two-dimensional (2D) images alone [7]. Brouwers et al. [7] showed that the use of 3D reconstruction modalities resulted in better understanding of acetabular fracture morphology and improved interobserver agreement on fracture classification between students and surgeons. Meesters et al. [8] found that different 3D reconstruction modalities could reduce operation time and intraoperative blood loss in acetabular fracture surgery. However, to the best of our knowledge, no research has been carried out on the influence of virtual 3D fracture reconstructions on the preoperative planning and the subsequent treatment strategy for the most challenging types of acetabular fractures, namely both-column acetabular fractures. Therefore, the aim of this study was to perform a nationwide study to determine (1) to what extent pelvic surgeons agree on the treatment strategy of both-column acetabular fractures based on conventional imaging (pelvic radiographs and 2DCT scans), and (2) whether the use of virtual 3D fracture reconstructions (in addition to conventional imaging) changes the surgeons’ preferred treatment strategy regarding the following three aspects: conservative vs. operative treatment, surgical approach, and surgeons’ level of confidence regarding the treatment strategy of choice.

## 2. Materials and Methods

### 2.1. Study Design and Participants

A nationwide cross-sectional survey study was performed in all trauma centers involved in pelvic fracture care in the Netherlands. Forty orthopedic trauma surgeons specialized in pelvic surgery were approached to participate in a series of two surveys: a survey containing 2D imaging data (2D survey) and subsequently a survey containing additional 3D imaging data (3D survey). Both surveys included a case description with clinical and radiological data of fifteen patients who were all treated in a level one trauma center and sustained a both-column acetabular fracture between 2007 and 2020. The availability of good-quality CT scans (slice thickness ≤ 2mm) was used as an inclusion criterion. In each survey, the surgeons were questioned on how they would treat the presented case based on the provided patient information and imaging. The surveys were built in Research Electronic Data Capture, a secure web application for building and managing online surveys and databases (REDCap, Paul Harris, Vanderbilt University Medical Centre). To prevent recall bias, the 3D survey was sent two weeks after completing the 2D survey and the same cases were presented in a different order.

### 2.2. ‘2D Survey’ about the Treatment Strategy of Both-Column Fractures Based on Pelvic Radiographs and 2DCT Images

**Figure 1:**
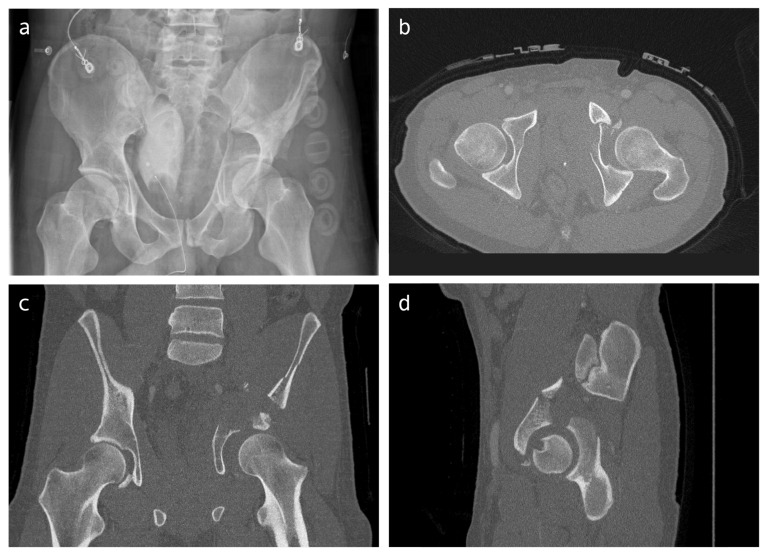
Conventional imaging provided within the surveys: (**a**) pelvic radiograph (AP overview), (**b**–**d**) examples of the axial, coronal, and sagittal CT slice (surgeons could scroll through every slice of the CT scan).

A ‘2D survey’ was generated to gain knowledge on how both-column acetabular fractures are currently treated. First, the surgeons were asked to answer a few general questions regarding their experience with pelvic surgery. They were asked (1) how many years of experience they have with pelvic surgery, and (2) how many acetabular fractures they operate on in a year. Next, they were asked to judge fifteen cases. For each case, basic patient information (gender and age), pelvic radiographs (anteroposterior (AP) overview (Figure 1a)), and 2DCT images (axial, coronal, and sagittal views (Figure 1b–d)) were provided. The images were retrieved from the electronic health record as DICOM images, were anonymized in Mimics Medical version 19.0 (Materialise, Leuven, Belgium), and uploaded to dicomlibrary: an online medical DICOM image file sharing service for educational and scientific purposes. It was possible to scroll through the CT slices and perform measurements in this viewer. For each case, the surgeons were asked to elaborate on which treatment they would recommend for the shown fracture: Nonoperative, Open Reduction and Internal Fixation (ORIF), or primary Total Hip Arthroplasty (THA). Surgeons were asked which surgical approach they would recommend when performing the surgery: anterior approach (either a Modified Stoppa approach possibly with a lateral window or an ilioinguinal approach), posterior approach (a Kocher–Langenbeck approach possibly with a trochanter osteotomy), or a combined approach (anterior as well as posterior), if ORIF was chosen. Finally, the level of confidence of the surgeon in the chosen treatment strategy (e.g., conservative versus operative and surgical approach) was given on a scale from 0 to 100 (zero indicating no confidence at all and one hundred as having full confidence).

### 2.3. ‘3D Survey’ about the Treatment Strategy of Both-Column Fractures Based on Additional Virtual 3D Fracture Reconstructions

**Figure 2:**
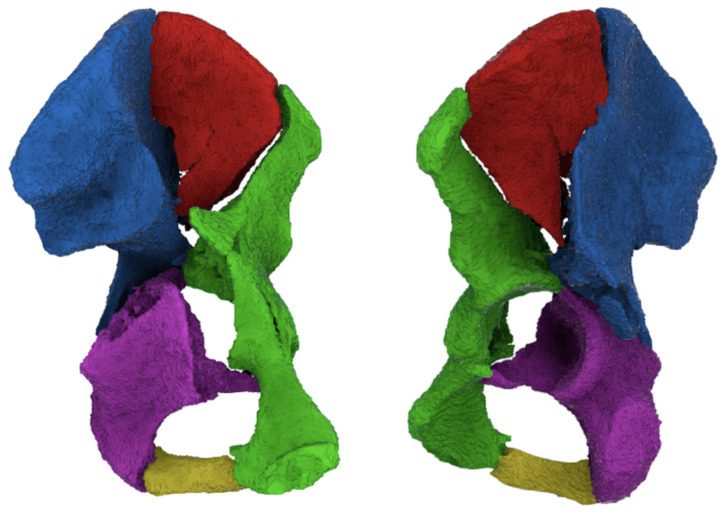
Virtual 3D reconstruction of a both-column acetabular fracture as provided within the surveys (surgeons could rotate and view the model in every direction). (**Left**): anterior view, (**right**): view into the acetabulum.

A ‘3D survey’ was generated to explore whether virtual 3D reconstructions of the fracture could influence the treatment strategy of both-column acetabular fractures. The same fifteen cases and case description information of the 2D survey were presented in this survey. Additionally, for each patient, the CT data (≤2 mm slices; spatial resolution, 0.5–0.6 mm) were transformed and used to create a 3D reconstruction using Mimics Medical software version 19.0 (Materialise, Leuven, Belgium) by an experienced technical physician (Figure 2). Semi-automatic segmentation of all fracture fragments was performed using a specific preset threshold for bone. Each fracture fragment was assigned a different color. The final 3D reconstructions were stereolithography (STL) files. These were exported to Filmbox (FBX) format and uploaded to Autodesk Viewer: a browser application that enables uploading, viewing, and sharing of 3D designs. It was possible to rotate the 3D model in all directions in this viewer. Surgeons were presented with the same information as in the 2D survey: age, gender, pelvic radiographs, and 2DCT images. However, in this 3D survey, a virtual 3D fracture reconstruction was presented in addition to the conventional imaging. Participating surgeons were asked the same questions as in the 2D survey: (1) elaborate on the preferred treatment strategy; (2) which surgical approach they would use to perform the surgery; and (3) the level of confidence in the chosen treatment strategy.

### 2.4. Statistical Analysis

Statistical analysis was performed with IBM SPSS Statistics for MacOs, version 27.0 and 28.0 (IBM Corp., Armonk, NY, USA). Characteristics of participating surgeons were summarized with medians and interquartile range (IQR) for not normally distributed variables. Categorical variables were presented as frequencies with percentages. Comparison of the treatment strategy based on pelvic radiographs and CT images, and the treatment strategy based on pelvic radiographs, CT images, and virtual 3D reconstructions, was performed with crosstabs and chi-square statistics. Differences were considered statistically significant with a *p*-value ≤ 0.05.

Interobserver agreement, defined as the level of agreement amongst the surgeons in either the 2D survey or the 3D survey, was calculated with Fleiss Kappa statistics. Interobserver agreement was calculated separately for each survey (2D/3D) for the treatment method and surgical approach. Kappa values were interpreted using the Altman guidelines adapted from Landis and Koch [9,10]. Kappa (κ) < 0.20 was defined as poor agreement; κ 0.21–0.41 as fair agreement; κ 0.41–0.60 as moderate agreement; 0.61–0.80 as substantial agreement; and 0.81–1.00 as almost perfect agreement. Level of confidence of the surgeon in their chosen treatment strategy was measured on a scale of 0–100. Level of confidence < 60 was defined as poor level of confidence, level of confidence ≥ 60 and < 80 was defined as moderate, and level of confidence ≥ 80 was defined as good level of confidence.

## 3. Results

### 3.1. Participating Pelvic Surgeons

Forty orthopedic trauma surgeons were invited to participate in this study, of whom twenty-four surgeons completed the first (2D) survey. After a median interval of 38 days (IQR 21–43 days), 20 surgeons also completed the second (3D) survey (response rate 50%). Calculations were performed on the treatment decisions of these 20 surgeons who completed both surveys. The four surgeons who did not complete the 3D survey (due to lack of time and interest) were excluded from further analysis. The included surgeons had a median of 9 years (IQR 6–20 years) of experience in pelvic surgery and operated on a median of 23 acetabular fractures (IQR 13–40 fractures) a year.

### 3.2. Treatment of Both-Column Fractures Based on Conventional Imaging (2D)

The 2D survey included 15 cases and the inclusion of 20 surgeons provided us with a total of 300 treatment decisions for the analysis of the treatment strategy for both-column fractures. In most treatment decisions (*N* = 267 (89%)), surgical management was recommended. In only a few cases (*N* = 33 (11%)), with minimal displacement, nonoperative treatment was considered (Table 1). In the case of surgical management, the anterior approach was recommended in 159 out of 246 (65%) of the treatment decisions. The posterior approach was recommended in only 21 out of 246 (8%) treatment decisions. A combined approach was recommended in 66 out of 246 (27%) treatment decisions. There was a fair amount of agreement between surgeons regarding the choice between conservative vs. operative treatment (κ = 0.35 (95% CI 0.31–0.38)). There was a poor level of agreement among surgeons regarding the surgical approach (κ = 0.05 (95% CI 0.001–0.10)). Surgeons reported a moderate level of confidence (level of confidence ≥ 60 and <80) in determining their recommended treatment strategy with the availability of solely pelvic radiographs and 2DCT images.

### 3.3. Treatment of Both-Column Fractures Based on Virtual 3D Fracture Reconstructions

Surgeons changed their recommendation regarding the choice between nonoperative and operative treatment in 32 out of 300 (11%) treatment decisions (*p* = 0.09) with the addition of 3D reconstructions to conventional imaging. Most changes in recommendations (22 out of 32, 7% of the total 300 treatment decisions) consisted of a recommendation for conservative treatment based on conventional imaging and a recommendation for operative treatment with the addition of virtual 3D reconstructions. Furthermore, 10 changes (3% of the total 300 treatment decisions) were made from operative treatment based on conventional imaging to conservative treatment when 3D reconstructions were added. The level of agreement on the choice between operative and nonoperative treatment decreased from fair with conventional imaging to poor agreement with virtual 3D reconstructions (κ = 0.19 (95% CI 0.152–0.23)).

For some cases the treatment method was conservative, and for some cases the operative treatment was THA both in the 2D and in the 3D survey (Table 1). Therefore, in 233 out of the 300 treatment decisions a surgical approach was chosen, because for the other cases conservative treatment or THA were chosen in the 2D or 3D survey or in both. Surgeons changed their preferred treatment regarding surgical approach in 86 out of 233 (37%) treatment decisions (*p* = 0.006) with the addition of 3D reconstructions of conventional imaging. Most changes occurred between the anterior and combined approach. Surgeons who initially preferred an anterior approach changed their preference to a combined approach with 3D reconstructions in 29 out of 146 treatment decisions (146 is the total amount of treatment decisions with an anterior approach, when conservative treatment and THA were excluded in both the 2D and 3D survey, Table 2 and Table S1). The surgical approach remained the same when adding 3D images in 147 treatment decisions, where the anterior approach was chosen in 115 treatment decisions, the posterior approach in 2 treatment decisions, and the combined approach in 30 treatment decisions. The level of agreement between surgeons on the recommendation for a surgical approach slightly increased with virtual 3D reconstructions; however, the level of agreement remained poor (κ = 0.13 (95% CI 0.08–0.17)).

The level of confidence remained the same when adding 3D images in 161 treatment decisions, it remained poor in 29 treatment decisions, it remained moderate in 67 treatment decisions, and it remained good in 65 treatment decisions. A change in the level of confidence of the surgeons in their recommended treatment strategy was seen in 139 out of 300 (46%) treatment decisions when adding virtual 3D reconstructions (*p* = 0.02). An overall rise in the level of confidence was observed and most changes (Table 3), 87 out of 300 treatment decisions (29%), were deemed positive (e.g., rise from poor level of confidence to either moderate or good level of confidence or rise from moderate to good level of confidence). However, we also observed a negative change in the level of confidence in 52 out of 300 (17%) treatment decisions. Yet, most negative changes (88% of a total of 52) were only one category of change when the level of confidence decreased, whereas 24% (of a total of 87) of the positive changes were from a poor to a good level of confidence.

## 4. Discussion

In this study, it has been shown that trauma/orthopedic surgeons agree to a large extent on the treatment method for both-column fractures, but to a far lesser extent on the surgical approach to both-column fractures. The addition of virtual 3D reconstructions influenced the surgeons’ preferred surgical approach and increased the surgeons’ level of confidence in twelve of the fifteen cases.

This study showed that both-column fractures are one of the fracture types that are mostly treated surgically, namely 89% (2D imaging) to 93% (3D imaging). This is in line with previous research by Audretsch et al. [11], where they found 85% surgical treatment. This study showed no statistically significant change in preferred treatment (conservative or operative), which is in contrast with the results of the study by Van den Berg et al. [12], who found that additional 3DCT reconstructions led to a higher percentage of surgical treatment on posterior tibial plateau fractures. This difference may be explained by the complex nature of both-column fractures and the high preference for surgical treatment that has already been established in the 2D survey with 89% surgical treatment. Therefore, virtual 3D reconstructions might enable better visualization and understanding of the fracture; however, 3D reconstructions do not necessarily change the probability of surgical treatment since there already is a high probability of surgical treatment.

In the present study, two separate cases (case 6 and 8) showed a remarkable increase in the preference for surgical management, of 30% and 35%, respectively. After reevaluation of these cases, this was likely due to an underestimation of the displacement of the fracture from conventional images (2D) in comparison with the 3D reconstructions, which evidently showed larger displacement than expected based on the 2DCT images. This indicates that 3D reconstructions can have an added value for determining the treatment method (surgical or conservative) in specific cases.

Audretsch et al. [11] stated that the lack of scientific data on how to approach acetabular fractures leads to therapeutic decisions mostly being based on the clinical experience of the surgeon. The choice of surgical approach mainly remains up for debate amongst surgeons and the results of this study affirm the statement by Audretsch et al. [11]. Different levels of training and (lack of) knowledge of different surgical approaches could be the reason why there is no clear decision making for what surgical approach would be the best fit for a specific fracture pattern. The influence of the addition of virtual 3D reconstructions to this process of preoperative planning is something that has not been researched before for acetabular fractures. Nonetheless, Halai et al. [13] showed that for calcaneal fractures the relatively less experienced surgeon benefited from 3DCT reconstructions of the fracture, and that the addition of virtual reconstructions led to a change in surgical approach in all cases regarding calcaneal fractures. For acetabular surgery, it is important to acknowledge the additional information on the acetabular fracture provided by the 3D reconstructions. A shift in surgical approach from anterior to posterior would mean an entirely different surgical preparation. Secondly, changing the surgical approach may lead to better visualization and therefore improve fracture reduction, which would ultimately improve overall the outcome. Finally, a shift from a combined approach to a single approach would mean less extensive surgery and therefore reduce the risk of complication during or after surgery. However, one should keep in mind that the changes in surgical approach seen in this study might not be solely due to the addition of 3D reconstructions but can simply be a result of the complexity of both-column fractures or a surgeon’s intra-observer variability.

This study showed that the addition of virtual 3D reconstructions increased the overall level of confidence of the surgeons in their treatment strategy. No clear explanation was found for the small number of treatment decisions in which a decline in the level of confidence was found. It is thought that this could be due to the experience of the surgeon with 3D reconstructions. When relatively inexperienced with 3D reconstructions, the addition might cause more confusion than clarification. Another explanation could be an underestimation of the complexity of the fracture from conventional imaging, where 3D reconstructions showed the actual extent of the fracture (more complex than expected) indicating more difficult reduction and the need for more extensive preoperative planning. Thus, it could be the case that the decline in confidence should not solely be attributed to the addition of 3D reconstructions, but also to the complexity of the fracture and the subsequent treatment strategy, which became more obvious. Despite this realization, 3D reconstructions will allow for better surgical preparation in advance and allow the surgeon not to be surprised by the extent of the fracture during surgery.

This study has some strengths and some limitations. First, this study focused only on both-column acetabular fractures. The reason to study only these complex fractures is that these fractures involve both columns, thus it is challenging to choose the optimal treatment strategy, including the surgical approach. Therefore, these fractures might benefit the most from virtual 3D reconstructions due to the improved visualization and understanding of the fracture, in contrast to simpler types of fractures. Additionally, including only both-column fractures resulted in a homogeneous study population. On the other hand, a limitation could be that our results mainly apply to both-column fractures and are not automatically generalizable to other types of acetabular fractures. Second, there was a response bias, because the response rate in this nationwide survey study was only 50%. The responding surgeons were all experienced in treating pelvic fractures and therefore we believe that our results are generalizable to the community of surgeons who treat acetabular fractures. Altogether, these surgeons evaluated 300 treatment decisions for both-column acetabular fractures both in 2D and in 3D and this number is sufficient to draw any conclusions about treatment variations for these injuries.

Virtual 3D reconstructions of both-column acetabular fractures may cause a shift in a surgeon’s preferred treatment strategy and improve the surgeon’s level of confidence in their treatment plan. Therefore, the usage of virtual 3D reconstructions is recommended for the treatment of both-column acetabular fractures. The development of evidence-based guidelines on surgical approaches for both-column fractures is needed because of the low level of agreement between surgeons, with a possible implementation of virtual 3D reconstructions in the diagnostic phase. The use of 3D imaging modalities may have added value in several phases of the treatment process [8]. First of all, 3D measurements can describe the severity of the fracture and be correlated with clinical outcome in terms of the eventual need for THA following acetabular fractures [14,15]. Likewise, 3D measurements can be correlated with total knee arthroplasty in tibial plateau fractures [16]. In our clinical practice, 3D virtual fracture models or 3D-printed models are already used in decision making regarding the treatment plan and preoperative planning. Three-dimensional printing might result in reduced operation time, less intraoperative blood loss, less intraoperative fluoroscopy time, and less postoperative complications [17,18,19,20]. Furthermore, 3D-printed models can be used for pre-contouring of the implant in order to optimize plate fitting [8]. Finally, patient-specific osteosynthesis plates and drilling guides could be used for complex fracture patterns [21], making it possible to determine the surgical approach and fixation techniques beforehand in a multidisciplinary team. Further research is recommended regarding the use of 3D imaging modalities for other types of acetabular fractures and possibly other types of fractures in general.

## 5. Conclusions

This study showed that surgeons agree to a lesser extent on the treatment strategy for both-column acetabular fractures, and mainly the type of surgical approach remains a subject of debate. The addition of virtual 3D reconstructions can influence a surgeon’s preferred treatment strategy. Moreover, virtual 3D reconstructions improve the level of confidence of the surgeon in their established method of treatment, from 38% reporting a good level of confidence (2D) to 50% reporting a good level of confidence (3D). Therefore, we conclude that virtual 3D reconstruction can aid in the assessment of complex fracture patterns, the amount of displacement, and in determining the treatment strategy in both-column acetabular fractures.

## Figures and Tables

**Table 1:** Recommended treatment method, surgical approach, and level of confidence for the treatment of both-column acetabular fractures.

		2D Survey	3D Survey
		N	N
Treatment method	Conservative	33 (11%)	21 (7%)
	Operative	267 (89%)	279 (93%)
Operative treatment specified	ORIF	246 (92%)	268 (96%)
	THA	21 (8%)	11 (4%)
Surgical approach	Anterior approach	159 (65%)	189 (71%)
	Posterior approach	21 (8%)	6 (2%)
	Combined approach	66 (27%)	73 (27%)
Level of confidence ^1^	Poor	64 (21%)	42 (14%)
	Moderate	121 (40%)	109 (36%)
	Good	115 (38%)	149 (50%)

^1^ Level of confidence: poor < 60, moderate ≥ 60 and <80, good ≥ 80.

**Table 2:** Number of changes (percentage) regarding surgical approach comparing only conventional imaging (2D survey) to the addition of virtual 3D reconstructions (3D survey). For instance, for 35 out of 233 treatment decisions (15%), the combined approach was chosen based on the 2D survey. Based on the 3D survey, the choice for a combined approach changed to an anterior approach in these 35 treatment decisions. Most changes occurred between the anterior and combined approach.

		3D Survey *		
		Anterior	Posterior	Combined
2D survey	Anterior	0 (0%)	2 (1%)	29 (12%)
	Posterior	11 (5%)	0 (0%)	8 (3%)
	Combined	35 (15%)	1 (0.4%)	0 (0%)

* The percentage is calculated based on the total of 233 treatment decisions for which a surgical approach was chosen.

**Table 3:** Number of changes (percentage) regarding surgeons’ level of confidence comparing only conventional imaging to the addition of virtual 3D reconstructions.

		3D Survey		
		Poor	Moderate	Good
2D survey	Poor	0 (0%)	18 (6%)	21 (7%)
	Moderate	12 (4%)	0 (0%)	48 (16%)
	Good	6 (2%)	34 (11%)	0 (0%)

## Data Availability

The authors declare that the data supporting the findings of this study are available within the paper.

## Supplementary Materials

The following supporting information can be downloaded at https://www.mdpi.com/article/10.3390/diagnostics13091629/s1, Table S1: Surgical approaches chosen in the 2D and 3D survey. Recommended surgical approach for treatment of both-column acetabular fractures based on conventional imaging and virtual 3D reconstructions.

## References

[1] AO Foundation AO Surgery Reference. https://surgeryreference.aofoundation.org/orthopedic-trauma/adult-trauma/acetabulum/both-columns.

[2] Kelly J., Ladurner A., Rickman M. (2020). Surgical Management of Acetabular Fractures—A Contemporary Literature Review. Injury.

[3] Mears D.C., Velyvis J.H., Chang C.-P. (2003). Displaced Acetabular Fractures Managed Operatively: Indicators of Outcome. Clin. Orthop. Relat. Res..

[4] Giannoudis P.V., Grotz M.R.W., Papakostidis C., Dinopoulos H. (2005). Operative Treatment of Displaced Fractures of the Acetabulum. A Meta-Analysis. J. Bone Joint Surg. Br..

[5] Giordano V., Acharya M.R., Pires R.E., Giannoudis P.V. (2020). Associated Both-Column Acetabular Fracture: An Overview of Operative Steps and Surgical Technique. J. Clin. Orthop. Trauma.

[6] Keel M.J.B., Siebenrock K.-A., Tannast M., Bastian J.D. (2018). The Pararectus Approach. JBJS Essent. Surg. Tech..

[7] Brouwers L., Pull ter Gunne A.F., de Jongh M.A.C., van der Heijden F.H.W.M., Leenen L.P.H., Spanjersberg W.R., van Helden S.H., Verbeek D.O., Bemelman M., Lansink K.W.W. (2018). The Value of 3D Printed Models in Understanding Acetabular Fractures. 3D Print. Addit. Manuf..

[8] Meesters A.M.L., Trouwborst N.M., Vries J.P.M.D., Kraeima J., Witjes M.J.H., Doornberg J.N., Reininga I.H.F., IJpma F.F.A., ten Duis K. (2021). Does 3D-Assisted Acetabular Fracture Surgery Improve Surgical Outcome and Physical Functioning?—A Systematic Review. J. Pers. Med..

[9] Landis J., Koch G. (1977). The Measurement of Observer Agreement for Categorical Data. Biometrics.

[10] Altman D. (1999). Practical Statistics for Medical Research.

[11] Audretsch C., Trulson A., Höch A., Herath S.C., Histing T., Küper M.A. (2022). Evaluation of Decision-Making in the Treatment of Acetabular Fractures. EFORT Open Rev..

[12] Van den Berg J., Struelens B., Nijs S., Hoekstra H. (2020). Value of Three-Dimensional Computed Tomography Reconstruction in the Treatment of Posterior Tibial Plateau Fractures. Knee.

[13] Halai M., Hester T., Buckley R.E. (2020). Does 3D CT Reconstruction Help the Surgeon to Preoperatively Assess Calcaneal Fractures?. Foot.

[14] Meesters A.M.L., Oldhoff M.G.E., Trouwborst N.M., Assink N., Kraeima J., Witjes M.J.H., de Vries J.P.P.M., ten Duis K., IJpma F.F.A. (2022). Quantitative Three-Dimensional Measurements of Acetabular Fracture Displacement Could Be Predictive for Native Hip Survivorship †. J. Pers. Med..

[15] Meesters A.M.L., Kraeima J., Banierink H., Slump C.H., de Vries J.P.P.M., ten Duis K., Witjes M.J.H., IJpma F.F.A. (2019). Introduction of a Three-Dimensional Computed Tomography Measurement Method for Acetabular Fractures. PLoS ONE.

[16] Assink N., Kraeima J., Meesters A.M.L., El Moumni M., Bosma E., Nijveldt R.J., van Helden S.H., de Vries J.P.P.M., Witjes M.J.H., IJpma F.F.A. (2022). 3D Assessment of Initial Fracture Displacement of Tibial Plateau Fractures Is Predictive for Risk on Conversion to Total Knee Arthroplasty at Long-Term Follow-Up. Eur. J. Trauma Emerg. Surg..

[17] Tu D.P., Yu Y.K., Liu Z., Zhang W.K., Fan X., Xu C. (2021). Three-Dimensional Printing Combined with Open Reduction and Internal Fixation versus Open Reduction and Internal Fixation in the Treatment of Acetabular Fractures:A Systematic Review and Meta-Analysis. Chin. J. Traumatol.-English Ed..

[18] Papotto G., Testa G., Mobilia G., Perez S., Dimartino S., Giardina S.M.C., Sessa G., Pavone V. (2021). Use of 3D Printing and Pre-Contouring Plate in the Surgical Planning of Acetabular Fractures: A Systematic Review. Orthop. Traumatol. Surg. Res..

[19] Cao J., Zhu H., Gao C. (2021). A Systematic Review and Meta-Analysis of 3D Printing Technology for the Treatment of Acetabular Fractures. Biomed Res. Int..

[20] Lee A.K.X., Lin T.L., Hsu C.J., Fong Y.C., Chen H.T., Tsai C.H. (2022). Three-Dimensional Printing and Fracture Mapping in Pelvic and Acetabular Fractures: A Systematic Review and Meta-Analysis. J. Clin. Med..

[21] IJpma F.F.A., Meesters A.M.L., Merema B.B.J., Duis K., De Vries J.P.M., Banierink H., Wendt K.W., Kraeima J., Witjes M.J.H. (2021). Feasibility of Imaging-Based 3-Dimensional Models to Design Patient-Specific Osteosynthesis Plates and Drilling Guides. JAMA Netw. Open.

