# Supplementary material for: Both-Column Acetabular Fractures: Does Surgical Approach Vary Based on Using Virtual 3D Reconstructions?
Source: Diagnostics (Basel). 2023 May 5;13(9):1629. doi: 10.3390/diagnostics13091629 (PMC10178242; doi:10.3390/diagnostics13091629)
Supplement: Supplementary file 1 [file diagnostics-13-01629-s001.zip › diagnostics-2371274-supplementary.pdf]

Supplementary Materials

Table S1. Surgical approaches chosen in the 2D and 3D survey. Recommended surgical approach for treatment of both column acetabular fractures based on conventional imaging and virtual 3D reconstructions.

	Surgical approach in 2D survey			Surgical approach in 3D survey		
	Anterior	Posterior	Combined	Anterior	Posterior	Combined
Case	% of total			% of total		
1	79	11	10	90	0	10
2	50	0	50	70	0	30
3	44	6	50	85	0	15
4	28	28	44	30	5	65
5	87	0	13	78	5	17
6	100	0	0	100	0	0
7	100	0	0	100	0	0
8	73	9	18	56	11	33
9	44	6	50	63	0	37
10	80	0	20	79	0	21
11	83	0	17	75	0	25
12	65	15	20	70	5	25
13	45	25	30	40	5	55
14	50	5	45	60	0	40
15	85	10	5	85	0	15
